# Contextual factors of self-regulation in children and adolescents with chronic diseases – a qualitative analysis

**DOI:** 10.1186/s12889-020-10056-1

**Published:** 2020-12-23

**Authors:** Cindy Höhn, Gloria Metzner, Edith Waldeck, Manuela Glattacker

**Affiliations:** 1 Section of Health Care Research and Rehabilitation Research, Institute of Medical Biometry and Statistics, Faculty of Medicine and Medical Center, University of Freiburg, Hugstetter Str. 49, 79106 Freiburg, Germany; 2 Deutsche Rentenversicherung Rheinland-Pfalz, Edelsteinklinik, Fachklinik für Kinder- und Jugendrehabilitation, Lindenstr. 48, 55758 Bruchweiler, Germany

**Keywords:** Self-regulation, Treatment representations, Contextual factors, Rehabilitation, ICF, Adolescents, Children, Qualitative analysis

## Abstract

**Background:**

In recent decades, the prevalence of chronic diseases in children and adolescents has increased significantly. Contextual factors play a central role in the self-regulation of chronic diseases. They influence illness and treatment representations, disease management, and health outcomes. While previous studies have investigated the influence of contextual factors on children’s beliefs about their illness, little is known about subjective contextual factors of treatment representations of children and adolescents with chronic diseases, especially in the context of rehabilitation. Therefore, the aim of this qualitative analysis was to examine the contextual factors reported by chronically ill children and adolescents in relation to their treatment representations. Furthermore, we aimed to assign the identified themes to classifications of environmental and personal contextual factors in the context of the International Classification of Functioning, Disability and Health (ICF).

**Methods:**

Between July and September 2018, semi-structured interviews were conducted with *N* = 13 children and adolescents in rehabilitation to explore their rehab-related treatment representations and associated contextual factors. The interviews started with an open narrative question about expectations and beliefs about rehabilitation, followed by further detailed questions. The interviews were recorded on audio tape, transcribed, and analysed using thematic content analysis.

**Results:**

Participants raised six themes associated with their rehab-related treatment representations that were interpreted as contextual factors: the living situation before rehabilitation, the idea of rehabilitation, previous solution attempts, rehab pre-experiences, information that the children and adolescents received from the clinic or sought themselves, and the assumed attitudes of their parents concerning rehabilitation. All the themes could be assigned to the classification of environmental and personal factors in the context of the ICF for children and youth.

**Conclusions:**

Although contextual factors have an important impact on self-regulation, little attention is paid to their investigation. Personal and environmental factors probably influence patients’ treatment representations in terms of expectations and concerns as well as emotions regarding the treatment. Considering contextual factors could lead to the more appropriate allocation of medical care and the better customisation of treatment.

**Supplementary Information:**

The online version contains supplementary material available at 10.1186/s12889-020-10056-1.

## Background

Chronic diseases are very common in children and adolescents. There is a wide range in reported prevalence rates of chronic health conditions in childhood between 0.22 and 44% due to the different concepts and operationalisations [1]. The extensive and representative “Study on the health of children and adolescents in Germany – KIGGS” found that according to their parents 16.2% of children and adolescents up to the age of 17 years had a chronic health problem [2].

Insufficient treatment of chronic diseases in childhood and adolescence often leads to their persistence, impairs development, and ultimately leads to lower quality of life and professional capacity in adulthood [3]. Therefore, medical rehabilitation for children and adolescents plays a significant role in the healthcare system in Germany, where chronically ill children and adolescents are treated by a multi-professional team in a multimodal approach [3–5]. Typical components of rehab treatment are, for example, psychological therapy, physio- and ergotherapy, and health education.

The International Classification of Functioning, Disability and Health: Children and Youth Version (ICF-CY) serves as the conceptual framework for rehabilitation [6]. The ICF-CY is based on a bio-psycho-social model and is used to describe health conditions as well as health-related conditions. The functioning and disability of a person is seen as a dynamic interaction between health conditions and contextual factors. The contextual factors are divided into environmental and personal factors. While environmental factors are further classified, personal factors are not yet classified because of the considerable socio-cultural differences that exist worldwide. However, there are national approaches to specifying them [7].

A theoretical approach that also highlights the influence of contextual factors on illness management is the Common-Sense Model of Self-Regulation (CSM) [8]. This framework describes how individuals who experience or anticipate a health threat select, initiate, and maintain behaviours to manage their illness. The CSM assumes that being faced with a health problem leads to the activation of memory structures and the development of cognitive and emotional representations of illness and treatment as well as of behavioural beliefs such as self-efficacy [9, 10]. These representations influence personal coping strategies, and those in turn health outcomes. Individuals appraise the success or failure of their self-regulatory actions and adjust their future coping strategies as well as their cognitive and emotional representations.

The CSM emphasises that self-regulation is not a process carried out in solitude but rather is one shaped by contextual factors [11]. Contextual factors have an impact on every aspect of the CSM: on illness and treatment representations, coping procedures, the appraisal of self-regulation, and the health outcome. Such contextual factors might include but are not limited to personal trait factors (biological and genetic factors, personality), personal factors in the sense of prior experience of the medical system [12], the input and expertise of others [11], and personal and cultural values, beliefs, and norms [13, 14]. Experiences within an individual’s family, circle of friends or even society at large affect the perception, knowledge, and expression of illness as well as health behaviour, for example whether individuals even seek help, what kind of help they seek, how they evaluate the treatment, and the relationship between families and professionals [15–17]. Health professionals can also influence patients‘cognitive beliefs, for example by giving a positive prognosis or telling them that the therapy is safe and effective [12, 18]. Information provided by physicians can modify the relation between maladaptive health beliefs resulting from earlier personal experience and illness representations [19]. Furthermore, social support as well as cultural beliefs and values also influence the emotional representation of illness and treatment [20, 21].

Regarding personal factors, studies have shown self-efficacy beliefs, gender, age, race, and prior treatment experience to have an influence on illness and treatment representations [22–25].

A number of studies have focused on contextual factors in self-regulation in children and adolescents. Personal illness experience, age, verbal intelligence, and socio-economic status have been identified as significant predictors of illness conceptualisation [26], and parents‘beliefs about a child’s illness or disability have been found to influence choice of treatment [17]. However, to our knowledge, contextual factors of treatment representations in children and adolescents have not yet been investigated.

As rehabilitation plays a significant role for chronically ill children and adolescents, the goal of our analysis was to explore the subjectively perceived contextual factors of rehab-related treatment representations of children and adolescents, and to assign those perceptions to the environmental factors of the ICF-CY [6] and the recommendations of personal factors of Grotkamp and colleagues [7].

## Methods

### Design and setting

We conducted a qualitative study to explore rehab-related treatment representations and associated contextual factors in children and adolescents in inpatient rehabilitation. The study was carried out between July and September 2018. Semi-structured interviews were conducted with children and adolescents at the rehabilitation clinic Edelsteinklinik. The study received approval by the ethics commission of the University of Freiburg, Germany (195/18). Written informed consent was obtained from participating children and adolescents and their parents.

### Participants

Approximately 2 weeks before the beginning of rehabilitation, eligible children and adolescents were called at home and informed about the study by the patient management of the clinic. If the children or adolescents and their parents showed interest in participating, they were asked to give their written informed consent when they arrived at the clinic. Inclusion criteria required the children and adolescents to be 12 to 17 years old. Participants were selected using a purposive sampling approach with the aim of maximising variation in the sample in terms of age, gender, and diagnosis.

### Procedure

The new developed interview guideline was based on previous research on treatment representations in adult rehabilitation patients and qualitative research about patients’ experiences of medical rehabilitation [27, 28]. The interview guideline is attached as supplementary file 1.

All face-to-face interviews were individual interviews, meaning that only the child or the adolescent and the interviewer were present during the interview. They were conducted on the day of the participant’s arrival or the following day. Both female interviewers were psychologists with practice experience in interviewing and conversation techniques.

To initiate the conversation with the child or the adolescents and introduce the theme, the interviewer told the participant a little bit about herself (name, occupation, place of employment) and about the background and objective of the study. Then, the participating children and adolescents were asked a few initial questions, e.g., about their age, reason for rehabilitation, and whether they were alone or with a relative in the rehab centre. After this entry into the conversation, the interview started with an open-ended narrative question about the expectations and beliefs of the child or adolescent concerning his or her beginning rehab treatment. This was followed by specific questions about process and outcome expectations, concerns, and emotions regarding rehab treatment that guided the narratives thematically. In addition, a number of questions addressed contextual aspects of treatment representations concerning general conditions before rehabilitation and the social environment.

All interviews were digitally audio-recorded and in addition some field notes were taken. Afterwards, the tape recordings were anonymised and transcribed verbatim by a transcription service company. The interviews were conducted and transcribed successively over 3 months so that data saturation could be monitored. After the interview, participants were asked to fill in a brief demographic questionnaire.

### Data analyses

To analyse the data we conducted a thematic content analysis using a combined deductive and inductive coding approach [29]. In terms of the deductive approach, the analysis was framed by the interview guideline and previous research, especially a questionnaire assessing treatment representations in adult rehabilitation patients [27], as well by the underlying theoretical concept, the CSM [8]. Additionally, an inductive approach was applied to identify themes in the dataset proceeding bottom-up, close to the spoken word of the children and adolescents. Sticking close to these statements, the researchers tried to take a step back from their theoretical knowledge in order to maintain reflexivity. Two researchers (CH, GM) analysed the first interview independently and developed initial codes bottom-up from the transcript text. The different sets of codes were discussed in terms of similarities, discrepancies, and how to proceed with the coding. After reconciliation and synthesis of the two sets of codes, both researchers analysed a second interview independently. After further discussions in the researcher team about the codings, each of the two researchers analysed the remaining interviews separately. Throughout multiple meetings and reviews the extracted themes and subthemes were specified, text paragraphs re-read, and codes refined to capture an accurate depiction of perspectives of children and adolescents. In cases of ambiguity or disagreement, a third person in the research team (MG) was called in to arbitrate. This recursive analysis process enabled a sustainable system of codes to be developed. Rules for carrying out the coding were defined to ensure interrater-reliability and transparency.

All data were organised and coded using the qualitative software MAXQDA (Version 12).

The demographic questionnaire was analysed with IBM SPSS Statistics 25. Information about the diagnoses of participants was documented by the physicians in the rehab clinic.

## Results

### Sample characteristics

Fifty children and adolescents as well as their parents were asked to agree to participate. While 37 refused without giving reasons, 13 children and adolescents (26%) could be interviewed. The interviews lasted between 20 and 40 min.

Participants were between 12 and 16 years old with a mean age of 14.3 years. Eight participants were male. Obesity was the most frequently reported primary diagnosis (*n* = 7). Other diagnoses (e.g., somatoform disorder, asthma, or chronic polyarthritis) were reported just once each. However, for eight of the 13 interviewees at least one comorbid diagnosis was reported. Two participants had been suffering from the disease for which they were in rehabilitation for less than 2 years, five children and adolescents reported a duration of between three and 5 years, and four participants a duration of more than 6 years. Two interviewees could not remember the duration of their disease. Three participants had been in rehabilitation before.

### Contextual factors of rehab-related treatment representations in children and adolescents

Throughout the successive conduction of the interviews, data saturation was monitored. We noticed highly recurring themes regarding the rehabilitation-related treatment representations, so we expected rarely new aspects with further interviews. The themes identified in the interviews could be assigned to the subjective rehab-related treatment representations of children and adolescents in the narrower sense reported elsewhere [30], as well as to variables that the children and adolescents associated with their treatment representations and which we interpreted as subjective contextual factors. A total of six subjectively significant contextual factors were identified and are presented below in more detail.

#### Living situation before rehabilitation

Besides the illness itself and its resulting limitations on activity and participation being reasons for rehabilitation, the living situation of the children and adolescents often influenced their motivation for rehabilitation and was reflected in corresponding outcome expectations. Interviewees described their living situation in terms of family life, school, and daily life prior to rehab treatment. They saw their family life as stressful, sometimes involving quarrels and the inability to be assertive. They had also experienced problems in school, reporting being unfocused in classes and getting bad marks and little respect from classmates. In addition, their daily life was delineated, for example by sleeping after school, spending a lot of time on the mobile phone, or having insufficient social contacts.

Interviewee: So I have a lot of stress at home, too, about school and stuff. I’m going to repeat the 9th grade because last year I lazed around a bit a lot and because of my weight I wasn’t always really concentrating at school. Yes, that made a lot of difference, whether I concentrate in class more now and get good grades or whether I’m not concentrating and get bad grades. (Participant - P01)

#### Idea for rehabilitation

The importance of the input and expertise of others as contextual factors was reflected in the fact that the idea for rehab treatment came from various persons in the social environment of children and adolescents. Participants reported that their parents, sister, physician, psychotherapist, youth welfare office, or youth care worker in the living group had suggested treatment. Sometimes the interviewees had the idea themselves; most of the time, they were joint decisions.

Interviewer: Who actually came up with the idea of going to rehab?

Interviewee: So my paediatrician just said, my mum also, that this might be quite good and then we looked. (P02)

#### Previous solution attempts

The impact of previous experiences on health behaviour and treatment decisions became clear when interviewees reported former attempts to find solutions, for example playing sports, finding a way to deal with painful symptoms, or joining an illness-related education programme. They also stated that they were regularly in outpatient care. However, previous attempts to find solutions had not been sufficiently successful and so their choice had now fallen upon rehabilitation.

Interviewee: We tried to get my weight back up by our own, but we couldn’t do it on our own and then they said that we would go to cure and that I would get help here. (P01)

#### Rehab pre-experience

Regarding prior experience of the medical system as a contextual factor for rehab-related treatment representations, interviewees mentioned their own rehab pre-experience and their satisfaction with it (e.g. success regarding weight loss, making new friends during the rehab stay). Former rehab experiences probably led to more knowledge of how rehabilitation works in general, which may in turn have influenced their expectations and concerns about this new rehab experience.

#### Information

Information, as a contextual factor that might also influence treatment beliefs, was found to be a relevant theme that could be divided into two subthemes: information from the clinic as a kind of input from others; and searches for information about the clinic by oneself. Statements by the children and adolescents on the subtheme of information from the clinic were quite heterogenous. While some reported having received no or at least some albeit insufficient information and expressed a wish for further information, others indicated that they had received sufficient information from the clinic before starting rehabilitation.

Interviewer: And did you get any information from the clinic?

Interviewee: Yes. Such a little booklet. There were a lot of things in it.

Interviewer: That means that you felt sufficiently informed in advance when you said that there was actually too much information?

Interviewee: Yes. I thought I was sufficiently informed. (P03)

Children and adolescents also mentioned searching for information about the clinic themselves, for example by asking family members who had already been in this rehab centre before or searching the Internet for information.

#### Assumed attitudes of the parents

Interviewees gave their perceptions of how their parents felt about their rehab stay and their parents’ probable expectations with regard to the process and outcome of rehabilitation. As a contextual factor, their parents’ attitudes may have shaped their own treatment representations.

Children and adolescents presumed that their parents had a positive attitude towards rehabilitation and viewed it as highly necessary.

Interviewer: And how important is it for your parents that you do rehab now, do you think?

Interviewee: I think that’s quite important to them, because otherwise they wouldn’t have said it like this, yes, o.k. well, doesn’t matter to us, so they’ve already said that’s important to them and that I’m also well, yes. (P01)

A few participants thought their parents had a critical attitude towards rehab stay, e.g. regarding the strict clinic rules or they had a generally negative attitude towards it.

Interviewee: They also found the thing with the mobile phone stupid, that it is taken away, because if they want to reach me or I them, if something happens or if something is that I just have my mobile phone. And they said they would see if it could be cancelled... (P04)

Interviewees also had perceptions about their parents‘concrete expectations of the process of rehabilitation. These focused on permanent care, daily school, and health education, as well as on hints for parents, e.g., recipes in the case of overweight children. Furthermore, some stated that their parents would expect them to be able to do things independently in the rehab centre and to get along with others.

Overall, children and adolescents believed that their parents had similar outcome expectations to their own. They perceived these to include better well-being, a reduction in physical symptoms, a change in health behaviour and greater independence. Furthermore, participants reported parental outcome expectations with regard to the consequences for daily life after rehabilitation, e.g., better use of leisure time (being more active, socialising with peers), improvement in school, and better interaction with others (fewer quarrels at home, being more open-minded). While most outcome expectations referred to the child or adolescent, one referred to a direct effect of rehabilitation on the parents themselves, as can be seen from the following quote:

Interviewer: Can you imagine what your parents would wish for you, what here... what should change for you here?

Interviewee: That it will be easier for them too, if I don’t have to take so much medicine now, go to the doctor and... (P05)

Besides describing the content of contextual factors, a further aim of this contribution was to assign the themes found in the interviews to categories of contextual factors, specifically the environmental factors of the ICF-CY or personal factors [6, 7]. Table 1 suggests this corresponding classification.

**Table 1:** Assignment of themes to environmental and personal factors

**Theme in the interview**	**Environmental factors** [10]
idea for rehabilitation	chapter 3: “support and relationships” e310 – immediate family e330 – people in positions of authority (e.g., teachers) e355 – health professionals e360 – other professionals (e.g., social workers)
information	
information from the clinic	chapter 1 “products and technology” e1300 – general products and technology for education
**Theme in the interview**	**Personal factors** [11]
living situation before rehabilitation	chapter 5 “situation and socio-economic / cultural factors” i510 - integration into the direct family and social environment chapter 4 “attitudes, basic skills, and behavioural habits” i430 – social competence i442 – media competence i459 – regeneration habits i465 – communication habits
idea for rehabilitation	chapter 4 “attitudes, basic skills, and behavioural habits” i436 – self-competence (empowerment)
previous solution attempts	chapter 6: “other health factors” i615 previous interventions
rehab pre-experience	chapter 6: “other health factors” i615 previous interventions
information	
search for information about the clinic by oneself	chapter 4 “attitudes, basic skills, and behavioural habits” i430 – social competence i436 – self-competence (empowerment) i442 – media competence
Assumed attitudes of the parents	
assumed parental belief of necessity	chapter 4 “attitudes, basic skills, and behavioural habits” i419 – attitude towards interventions and technical aids
assumed parental process expectations	chapter 4 “attitudes, basic skills, and behavioural habits” i419 – attitude towards interventions and technical aids
assumed parental outcome expectations	chapter 4 “attitudes, basic skills, and behavioural habits” i419 – attitude towards interventions and technical aids

The theme “idea for rehabilitation” included personal as well as environmental aspects and was therefore assigned to both categories. With regard to personal factors, developing the idea of rehabilitation by oneself could be understood as part of self-competence in terms of self-management. This reflects, for example, the ability to represent personal goals in a self-responsible and self-determined manner. Regarding environmental factors, input from family members or health professionals, for example, illustrates the support by others at the beginning of the coping process.

The theme “information” also comprised personal and environmental factors. Searching for information by oneself reflected various personal competences. Besides self-competence, social competence in terms of skills for social interaction and the competence to discern the attitudes of others played a central role for those interviewees who asked family members about their experiences of rehabilitation. Furthermore, media competence (use of media for one’s own needs and goals) was relevant to those who searched the Internet for information. Getting information from the clinic as an environmental factor could be assigned to “general products and technology for education”, which include books and pamphlets to disseminate information.

The living situation before rehabilitation comprised information about integration within the family and social environment, for example having little contact with parents or peers, as well as information about personal skills and behavioural habits. In our sample, insufficient social and media competence (e.g. spending more time in virtual than in real life) and problematic regeneration and communication habits (e.g. sleeping a lot during the day and few communication with family and friends) became apparent. Pre-experiences of rehab as previously attempted solutions were assigned to “previous interventions”. The assumed attitudes of parents were assigned to the personal factor “attitude towards interventions and technical aids”. According to Grotkamp et al. this factor comprises personal beliefs in connection with accepting a treatment [7]. This might also include perceived parental beliefs about the necessity of and expectations about treatment.

## Discussion

In our qualitative analysis, children and adolescents reported a range of perceived contextual factors associated with their rehab-related treatment representations. These contextual factors could be mapped to the classification of environmental and personal factors in the context of the ICF-CY [6, 7].

Two important environmental factors emerged: namely, the idea for rehabilitation and information from the clinic. Corresponding to the notion that illness management occurs in a social context, the idea of undergoing rehab treatment could be encoded to the “support and relationships” chapter of the ICF-CY [6]. The idea for rehabilitation usually came from various persons, as stated in the interviews, illustrating the shared responsibility for managing the illnesses of children and adolescents. On the one hand, children and adolescents in the interviewed age group were increasingly taking on responsibility for managing their own illness; on the other, illness management in this age group could still be shared between parents and the children, especially with the younger patients in our sample. Consequently, Sonney and Insel have argued for a reformulation of the CSM in paediatric care [31]. They suggest a common-sense model of parent-child shared regulation that includes parent and child representations, both of which influence the coping procedure and management of the disease.

Furthermore, information as an environmental factor (the “general products and technology for education” chapter) was shown to have an impact on the self-regulation process and thus on individual representations of illness and treatment. In this regard, Glattacker, Heyduck and Meffert showed that information adapted to patients’ illnesses and treatment perceptions, as well as to their information needs, leads to greater perceived personal control as part of illness representations [32]. Moreover, information could help to prevent or correct false expectations [19].

Themes identified in the interview data that could be ascribed to personal factors included the living situation before rehabilitation, the idea for rehabilitation, previous attempts to find solutions, rehab pre-experiences, searches for information about the clinic by oneself, and the assumed attitudes of parents. These themes could be encoded to the categories “situation and socio-economic / cultural factors”, “attitudes, basic skills, and behavioural habits”, and “other health factors” proposed by Grotkamp et al. [7]

Statements in the interviews about the living situation before rehabilitation comprised aspects such as stress at home, problems at school, and a lack of social contact that could increase interviewees’ motivation to go into rehabilitation. Moreover, these aspects could influence process and outcome expectations (e.g., stress reduction, improvement in school, meeting new friends), conceptualised as dimensions of rehab-related treatment representations in adolescents [30].

The idea for rehabilitation is not only an environmental factor in terms of the input of others but it is also a personal factor that demonstrates the self-competence of the adolescent in developing the idea for treatment by him−/herself. Self-competence in terms of self-management probably has a strong influence on self-regulation.

Previous attempts to find a solution could influence emotional and cognitive treatment representations too, and in both directions: patients with previously unsuccessful attempts might be either especially hopeful that rehab treatment finally helps or sceptical because they assume that it will not help either. Accordingly, representations are further influenced by previous rehab treatment experiences. Children and adolescents who had already been in rehabilitation reported that they had presumptions about the rehab treatment. Sonney and Insel have argued that past experiences shape future self-regulation [31]. Having a history of former treatment might lead to more accurate expectations [23].

A further relevant contextual factor was the childrens’ or adolescents’ perceptions of the parents’ treatment representations. Our interviewees highlighted the necessity and importance of rehabilitation for their parents. Furthermore, the process and outcome expectations they attributed to their parents were similar to their own [30]. Research shows that in children and adolescents, carer beliefs have notable effects on treatment adherence and outcomes [33, 34]. Parental beliefs might also shape the illness representations of their children, while the extent of the match between the illness representations of parents and of adolescents might influence adolescents’ self-management [35]. In Germany, it is possible for children and adolescents in rehabilitation to be accompanied by a family member. This not only allows to address illness and treatment representations of children and adolescents but also parents’ representations, and to empowers family supporters with the aim of improved patient self-management and better health outcomes [36].

### Limitations

One limitation of the study is the selectivity of our sample. Only a quarter of children and adolescents who were asked to participate actually agreed to take part. Those who did not may have differed from those who did on subjectively important contextual factors. Due to the high number of children and adolescents with a primary diagnosis of obesity in the rehab centre, the range of primary diagnoses within our sample was also limited. Nevertheless, other diagnoses were represented as comorbid diseases. Furthermore, in rehabilitation the priority is given not to diagnoses but to the restrictions on functioning they incur. In addition, it should be stated that we only presented contextual factors of rehab-related treatment representations that were reported by the children and adolescents in the interviews. Given the variety of possible environmental and personal factors [6, 7], further important contextual factors may influence the self-regulation process. Interpreting our results, it should be taken into account that the generalizability of our results to other age groups such as younger children is limited. We decided to interview older children and adolescents at the age of 12 to 17 years. This lifespan is very dynamic due to biopsychosocial changes. This might result in reporting other subjectively important contextual factors for treatment representations than would have been the case in younger children. However, we chose this age group because treatment representations are abstract constructs that can be hardly verbalized at younger age – keeping in mind the developmental stages of abstract thinking. Furthermore, for children younger than 12 years surveys in rehabilitation are commonly filled in by their parents. A further limitation is that we did not conduct any member checking of the results.

### Implications for further research and practice

For children and adolescents in particular, further studies should consider a broader social environment to clarify whose input works as a contextual factor of treatment representations, as this may be particularly relevant in this age group. In adolescence, the influence of peers and social media might be more pronounced. Additionally, practitioners can also influence patients’ beliefs and health outcomes and should therefore be examined further in future research [18, 21]. Such associations should preferably be explored in larger samples and with a different, for example quantitative, methodology.

For parental beliefs, we asked the children rather than the parents themselves. To what extent actual parental expectations and childrens’ or adolescents’ perceptions of their parents’ beliefs match, as well as what impact they have on the self-regulation process of children and adolescents, should be investigated in further studies.

With regard to clinical practice, a central recommendation might be to pay more attention to exploring subjective contextual factors. Such variables might serve as facilitating factors as well as barriers to self-regulation and could be modified in a positive way, for example, individually adapted information and brief psychological interventions could optimise patients’ expectations, minimise unrealistic expectations, and ultimately lead to better health outcomes [32, 37]. Furthermore, considering personal contextual factors such as attitudes, competences, and habits might lead to more appropriate allocation of medical care and customised treatment [38].

## Conclusions

The development, maintenance, and change of expectations as an underlying component of treatment representations [37] takes place in a dynamic process that is influenced by contextual factors [12]. Although contextual factors are an important element of the CSM, studies of this framework have to date often failed either to include or focus on this aspect [39]. The present qualitative analysis gives some first indications of relevant contextual factors regarding rehab-related treatment representations as reported by children and adolescents in inpatient rehabilitation. This field of research deserves further attention because contextual factors are relevant for self-regulation, especially in dealing with chronic diseases that occur more and more frequently in children and adolescents. Contextual factors may act as facilitating factors mitigating the negative effects on functioning of a health problem (e.g., the optimistic beliefs of professionals) as well as barriers that hinder participation (e.g., the poor accessibility of health service providers). However, contextual factors and their influencing potential must be seen from an individual point of view, because the same contextual factor might be experienced as facilitative or aggravating depending on the person’s health condition.

## Supplementary Information

**Additional file 1.** Interview guideline.

## Data Availability

The datasets generated and analysed during the current study are not publicly available due to data privacy reasons. Primary data (interviews) are not anonymous due to the voice of the participants which allows identification. Even pseudonymised transcribed data might allow identification by a combination of the personal information reported by the adolescent (e.g. family situation, kind of chronic disease, medical pre-experiences) and the time of data collection.

## Abbreviations

KIGGS: Study on the health of children and adolescents in Germany
ICF-CY: International Classification of Functioning, Disability and Health: Children and Youth Version
CSM: Common-Sense Model of Self-Regulation
P: Participant
