# Supplementary material for: Contextual factors of self-regulation in children and adolescents with chronic diseases – a qualitative analysis
Source: BMC Public Health. 2020 Dec 23;20:1923. doi: 10.1186/s12889-020-10056-1 (PMC7758920; doi:10.1186/s12889-020-10056-1)
Supplement: Supplementary file 1 — Additional file 1. Interview guideline. [file 12889_2020_10056_MOESM1_ESM.docx]

Supplementary file 1: Interview guideline

**Research project**

**“Predictors of patient satisfaction in the rehabilitation of children and adolescents" - a project of the rehabilitation scientific research association**

INDIVIDUAL INTERVIEWS

**INTRODUCTION**

| 1. The interviewer  a) introduces herself (information about profession, work place, research project)  b) thanks the child or adolescent for participation in the interview study  c) informs the child or adolescent about:   - the content of the interview (personal view on rehabilitation) - the duration of the interview (30-45 minutes) - that the medical staff is not told about what the interviewee reports - the recording of the interview on tape - data privacy   2. The participant should introduce himself/herself briefly, e.g. age, reason for rehabilitation  3. Switching on the tape recorder (recording ID number) |
| --- |

**INTERVIEW GUIDELINE**

*Entry into the interview*

| **Idea for rehabilitation/ information / importance of rehabilitation** |
| --- |
| - **Who had the idea for rehabilitation?**   If someone else had the idea:   - - What did you think about the idea of rehabilitation?   - What spoke for or against going to rehabilitation?   - Why do you think the person who recommended rehabilitation believes that rehabilitation is good for you?   - What do you think, how important is it for your parents that you are in rehabilitation? - **How did you feel when you arrived in the rehabilitation clinic?** (Were you rather sceptical or hopeful?) - **Do you know what rehabilitation is?**   - Have you received any information in advance about what rehabilitation is?   - Do you feel sufficiently informed or would you have wished further/other information in advance?   - Do you know which therapies the clinic offers? |

*Main part: Open narrative*

| **Open-ended question: Beliefs and expectations regarding rehabilitation** |
| --- |
| - **I am interested in your personal ideas about rehabilitation. You can just talk freely how you imagine the rehabilitation will be.** - What do you wish, that should happen here? What would you find good? What won’t you find good? |

*Questions for more detailed information*

| **Expectations regarding the process** |
| --- |
| - **How do you imagine a daily routine in rehabilitation? What will you do here from morning till night?** - What would be important for you in the daily routine in rehabilitation?   (e.g. regarding getting up, meals, therapies: individual or group therapy, age-, gender- or disease-specific therapy, frequency of therapy, compulsory attendance, clinic rules, clinic school)   - **How do you imagine the free time in rehabilitation? What will you do in your free time (between/after the therapies)? What do you expect how the weekends will be?** - **If the child or adolescent is accompanied by a parent:** - How do you imagine the rehabilitation together with your mother/father? - What do you expect, how much time will you spend together and what you will do together? (therapies / leisure time) - What do you like about the fact that your mother/father accompanies you and what don’t you like? - What do you expect from the accompaniment of your mother/father? - **How do you imagine the contact to your family / friends at home during rehabilitation?** - **How do you imagine the contact to other children / adolescents in rehabilitation?** - **What do you think would make your stay in rehabilitation easier / harder?** - What would lead to (dis-)satisfaction with rehabilitation? - **How important is it to you to participate in the development of your treatment plan?** - **How important is it to you to participate in setting your rehabilitation goals?** - if the child or adolescent is accompanied by a parent: How important is it to you that your mother/father is involved in the treatment planning and the setting of your rehabilitation goals? - **How important is it for you to have a dedicated contact person in the clinic and what do you want to use this person for?** |

| **Outcome expectations** |
| --- |
| - **What would you say: Why is rehabilitation necessary for you?** - **Which changes do you expect due to rehabilitation?** - **What do you think which changes do your parents expect due to rehabilitation?** - **Imagine the end of rehabilitation:** - How will you feel physically? - How will your mood be? How will the rehabiliation stay affect your everyday life/school/leisure time? - What do you expect to have learned? Do you think that you will deal with your illness differently after rehabilitation? If yes, how will you deal with your illness differently? - **Which changes due to rehabilitation do you expect for other people, e.g. your family?** - **Do you have a picture or a metaphor what rehabilitation means to you?** |

| **Concerns** |
| --- |
| - **Do you have any concerns that you associate with rehabilitation?**   (e.g. negative reactions from friends / schoolmates, homesickness, long absence from school, rehabilitation becomes strenuous, do not understand with other children or adolescents / do not make friends) |

| **Changed expectations** |
| --- |
| - **Before you arrived in the clinic, did you have any other expectations at home that we haven't talked about yet?** |

| **Patient satisfaction** |
| --- |
| - **Most children and adolescents are satisfied with their rehabilitation, but some are not. Could you imagine why some children / adolescents are dissatisfied with rehabilitation?** |

**END OF THE INTERVIEW**

| *Outro:*  Now we are at the end of the interview. Are there certain parts of the interview that you would like to have deleted afterwards? Thank you very much for your participation! All the best and a good stay. |
| --- |
